# Evaluation of the ankle function after Achilles tendon resection: a retrospective clinical study

**DOI:** 10.1007/s00402-023-05177-2

**Published:** 2024-01-17

**Authors:** Olimpiu Bota, Leona M. Heinzinger, Bianka Herzog, Alexander C. Disch, Michael Amlang, Philipp Flößel, Adrian Dragu, Feras Taqatqeh

**Affiliations:** 1 University Center for Orthopedics, Trauma and Plastic Surgery, Faculty of Medicine Carl Gustav Carus, TU Dresden, Fetscherstraße 74, 01307 Dresden, Germany; 2 Department of Plastic Surgery, First Surgical Clinic, Emergency County Hospital Cluj-Napoca, Iuliu Haţieganu University of Medicine and Pharmacy, Cluj-Napoca, Romania; 3 University Center for Orthopedics, Trauma and Plastic Surgery, Section Sports Medicine and Rehabilitation, Faculty of Medicine Carl Gustav Carus, TU Dresden, Dresden, Germany

**Keywords:** Achilles tendon infection, Achilles tendon reconstruction, Achilles tendon repair, Foot and ankle surgery, Reconstructive surgery, Negative pressure wound therapy

## Abstract

**Introduction:**

The Achilles tendon is the strongest tendon in the human body and has the function of plantar ankle flexion. When the tendon is exposed, the peritendineum has been breached and the thick avascular tendon colonized with bacteria, a complete resection of the tendon may be indicated to achieve infection control and facilitate wound closure. The Achilles tendon reconstruction is not mandatory, as the plantar flexion of the ankle joint is assumed by the remaining flexor hallucis longus, flexor digitorum longus and tibialis posterior muscles. Our study aimed to evaluate the impact of Achilles tendon resection without reconstruction on leg function and quality of life.

**Material and methods:**

We retrospectively evaluated all patients who were treated with an Achilles tendon resection between January 2017 and June 2022 in our quaternary institution. After evaluating the data, the patients who survived and were not amputated were contacted for re-evaluation, which included isokinetic strength measurement of both ankle joints, evaluation of the ankle range of motion and collection of several functional scores.

**Results:**

Thirty patients were included in the retrospective study, with a mean age of 70.3 years, including 11 women and 19 men. The most frequent cause of the infection was leg ulcer (43.3%), followed by open tendon suture (23.3%). No tendon reconstruction was performed. Fifteen patients could be gained for reevaluation. The average difference in ankle flexion torque on the injured side compared to the healthy side at 30 degrees/second was 57.49% (*p* = 0.003) and at 120 degrees/second was 53.13% (*p* = 0.050) while the difference in power was 45.77% (*p* = 0.025) at 30 degrees/second and 38.08% (*p* = 0.423) at 120 degrees/second. The follow-up time was between 4 and 49 months and a positive correlation could be determined between the time elapsed from surgery and the ankle joint strength. There was a significant loss of range of motion on the operated side compared to the healthy side: 37.30% for plantar flexion, 24.56% for dorsal extension, 27.79% for pronation and 24.99% for supination. The average Lepillhati Score was 68.33, while the average American Orthopedic Foot and Ankle Score was 74.53.

**Conclusion:**

The complete Achilles tendon resection leaves the patient with satisfactory leg function and an almost normal gait. Especially in elderly, multimorbid patients, straightforward tendon resection and wound closure provide fast infection control with acceptable long-term results. Further prospective studies should compare the ankle function and gait in patients with and without Achilles tendon reconstruction after complete resection.

## Background

The Achilles tendon (AT) is the strongest in the human body. Its contributing muscles are the medial and the lateral gastrocnemius and the soleus muscles, which together form the triceps surae muscle [1]. The AT function is plantar flexion of the ankle joint. As opposed to the mythological figure Achilles, the AT is not of vital importance to humans and when resected, the flexor hallucis longus (FHL), flexor digitorum longus (FDL) and tibialis posterior muscles can take over its function and act as plantar flexors of the ankle joint [3].

**Fig. 1:**
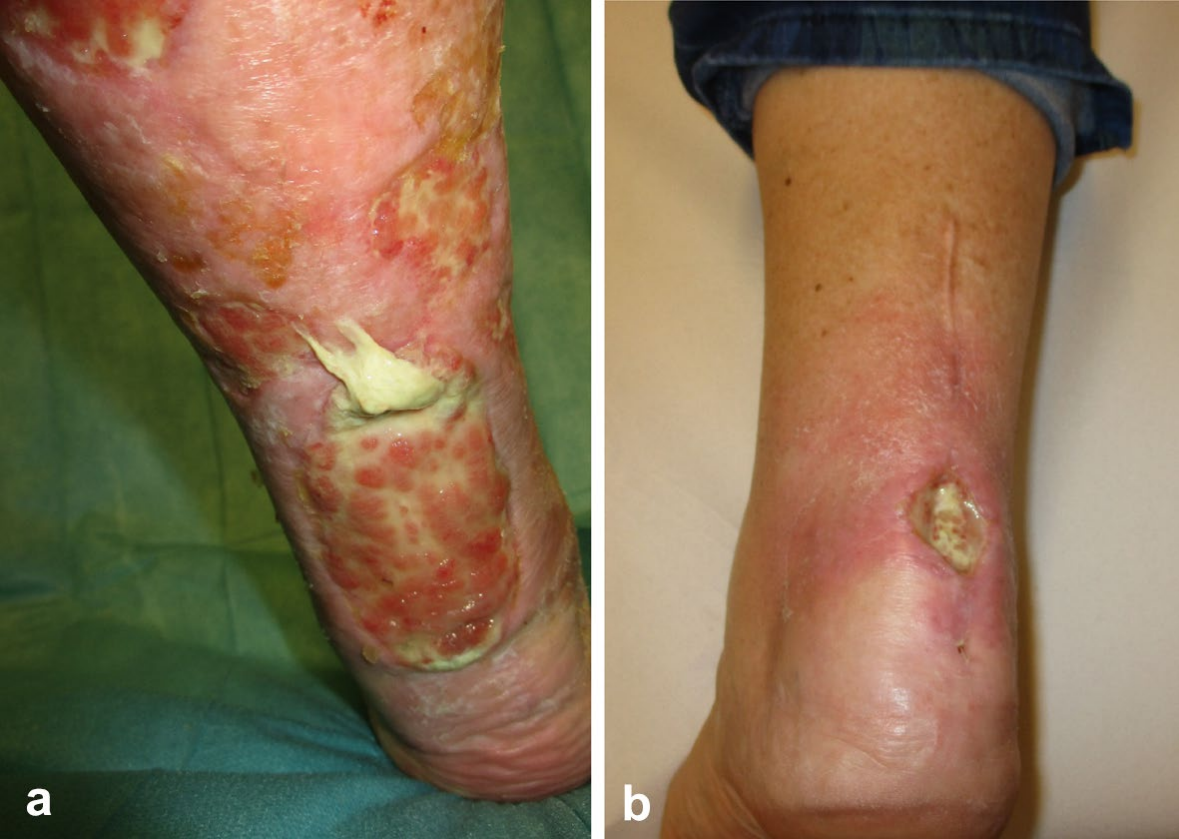
**a** Leg ulcer with necrotic AT. **b** Deep infection after open AT repair

Because the AT lies superficial, without muscle coverage, it can easily become exposed and infected. Blunt or sharp trauma, corticoid infiltration, surgical repair (Fig. 1b), leg ulcers (Fig. 1a) and other dermatological disorders associated with skin necrosis, all can lead to the infection of the tendon [4]. Early debridement and removal of necrotic tissues, foreign bodies, suture material and bone anchors are prerequisites to infection control [5]. Once the peritendineum has been breached and the thick avascular tendon becomes imbibed with water and colonized with bacteria, a complete resection of the tendon may be indicated to achieve infection control and facilitate wound closure [3, 4, 6].

After surgical debridement with radical Achilles tendon resection (ATR), efficient wound closure is necessary [7]. As many of these patients are multimorbid with tissue perfusion disorders of the lower extremities, a straightforward solution like direct skin suture or split-thickness skin grafting is often preferable to a complex free tissue transfer procedure with concomitant tendon reconstruction [4]. The goal of our study was to evaluate the residual function of the ankle joint and the quality of life in patients after Achilles tendon resection. So far there is to our knowledge only one publication in the literature, which evaluates the function of seven patients after ATR.

## Material and methods

**Fig. 2:**
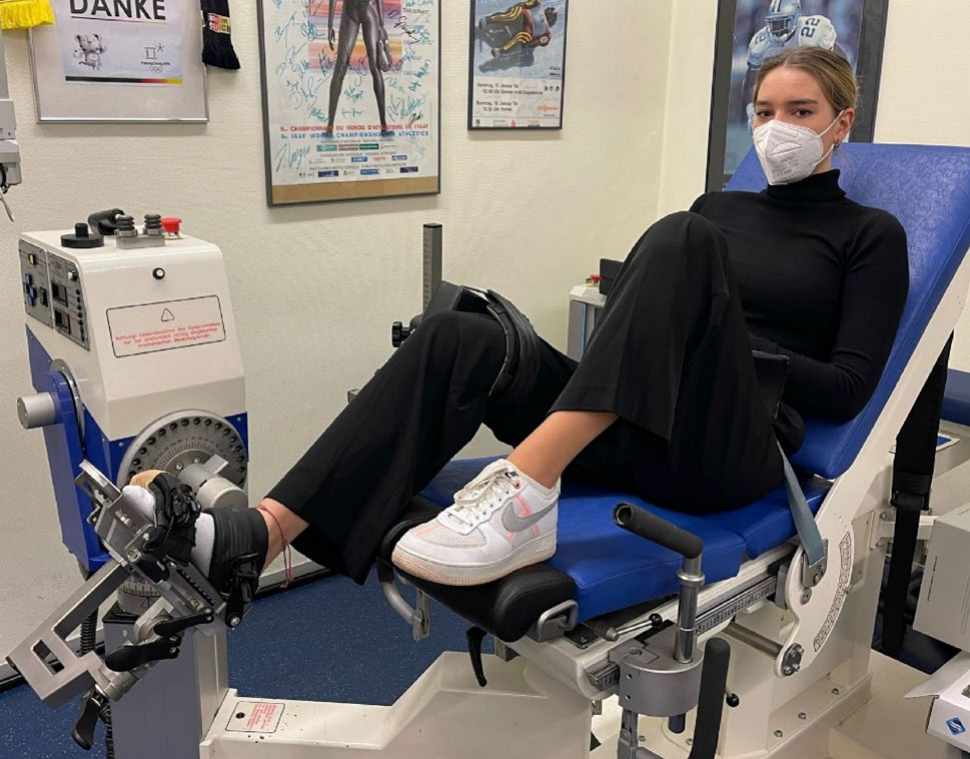
IsoMed 2000® device with healthy volunteer ready for ankle joint strength measuring

We retrospectively gathered the data from all patients who were treated with an Achilles tendon resection between January 2017 and June 2022 in our quaternary care institution. After evaluating the data, the patients who survived and were not amputated were contacted for the second part of the study. Six patients (20%) died in the meantime (Table 1), six patients refused to take part in the study and one patient could not be reached. Fifteen patients could present themselves in our clinic for the study. After signing the informed consent, the patients were clinically examined and three ankle and hindfoot specific scores were ascertained: the Achilles Tendon Total Rupture Score (ATRS), which includes 10 questions for the patient to answer, the American Orthopedic Foot and Ankle Score (AOFAS), a commonly used score which combines a patient-reported and a clinician-reported part, and the Leppilhati Score, which is composed from 5 subjective and 2 objective questions. Furthermore, two more scores were collected:the Visual Analog Scale (VAS), where the patients report their level of pain on a scala from 0 to 10 and the EuroQol 5Dscore, where the patients evaluate their quality of life and health status.. The range of motion of both ankle joints was measured (plantar flexion/dorsal extension and pronation/supination). An isokinetic strength measurement of both ankle joints was performed, using an angular velocity of 30 degrees per second (°/s) and 120°/s with the device IsoMed 2000® (D. & R. Ferstl GmbH, Hemau, Germany) (Fig. 2). Two measurements were performed, each with three repetitions. The better of the two measurements was used and an average was made from the three obtained values. One patient had a poor general condition and could not perform the force measurement. Another patient could perform all the measurements, except for the 120°/s on the operated leg. The average follow-up time was 19 months (4–49 months).

**Table 1:** Functional scores

Score	Average	Maximum
ATR	47,27	100
AOFAS	74,53	100
Lepillahti	68,33	100
VAS	2,07	10
EuroQuol	55	100

The data were gathered in Excel 365 (Microsoft Corporation, WA, USA). For the statistical analysis JASP version 0.16.4 (University of Amsterdam, The Netherlands) was used. Normal distribution was tested with the Shapiro–Wilk test. Numerical non-matched variables were tested with the t-test if normally distributed or with the Mann–Whitney U-test otherwise. For matched dependent variables, the Wilcoxon signed-rank test was used. Categorical variables were compared using Fisher´s Exact test. Correlations were analyzed using Pearson’s correlation coefficient for normal distributed variables, while for the non-normal distributed variables Spearman’s rank correlation coefficient was employed. Statistical significance was considered when *p* < 0.05.

The study was approved by the institutional review board (BO-EK-196032021). A signed informed consent was obtained for the reevaluation.

### Radical ATR—operative technique

**Fig. 3:**
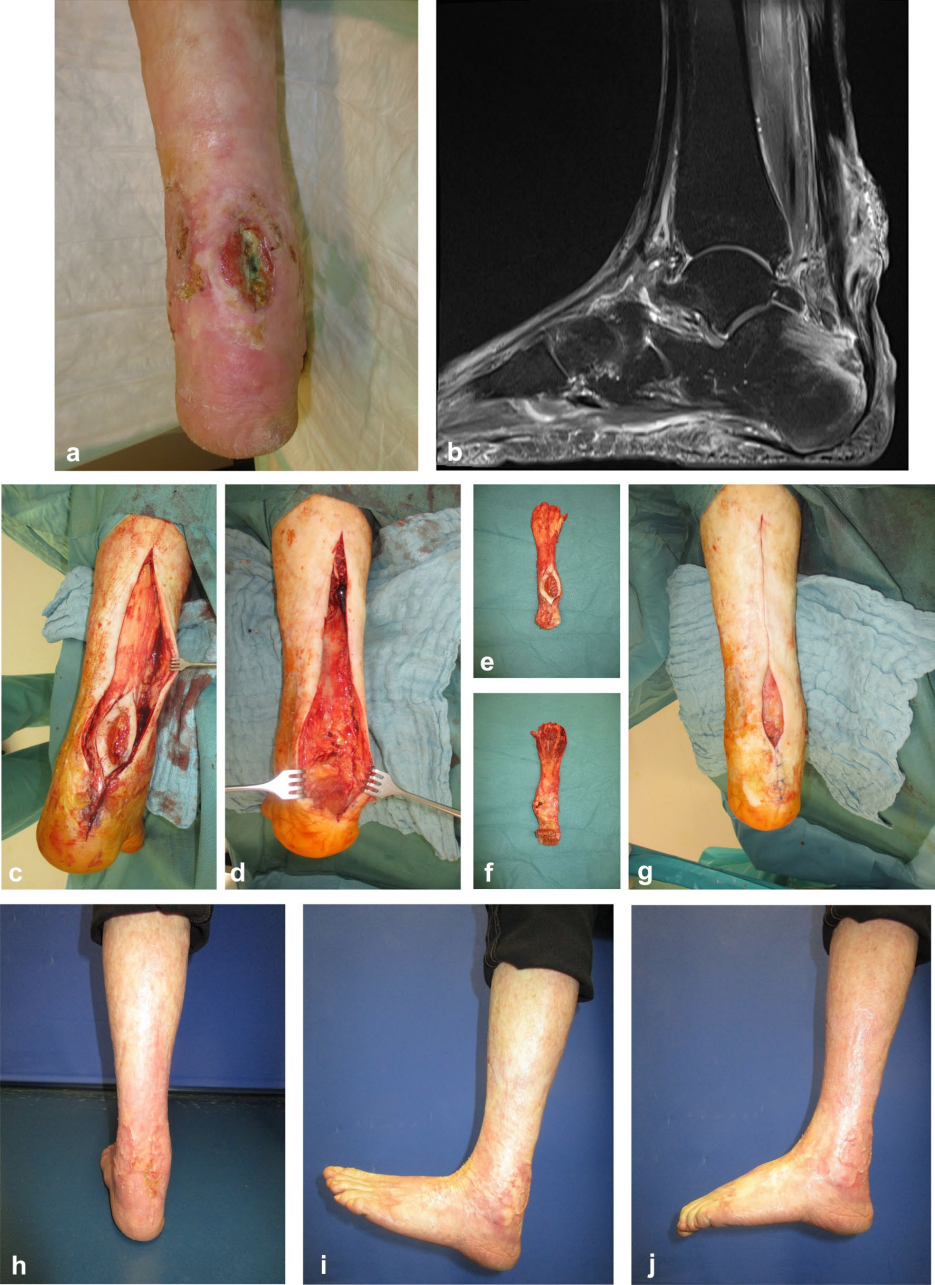
Case report 1: 58-year-old male with graft-versus-host disease and AT infection after R0 resection of a cutaneous squamous cell carcinoma. **a** Initial finding. **b** MRI shows osteomyelitis of Tuber calcanei. **c** Tendon prepared to be resected in one piece. **d** Surgical wound after en bloc resection of the AT with Tuber calcanei. **e** Resected specimen, posterior view. **f** Resected specimen, anterior view. **g** Leg after primary wound closure; a 50 × 15 mm remains for NPWT. **h** Final result three months postoperative after split-thickness skin grafting. **i** Dorsal extension of the ankle joint. **j** Plantar flexion of the ankle joint

The indication for ATR is given when the exposed Achilles tendon with breached peritendineum shows imbibition either clinically or by MRI. A failed wound closure with infection relapse or bone involvement also indicates an ATR. The surgery is performed in supine position or lateral decubitus, with the diseased leg lying on top of the healthy one. Regional anesthesia is preferred whenever possible. The incision encompasses the existing wound and extends along the posterior midline upwards until the tendon–muscle junction is reached and downwards to the Tuber calcanei. The AT is prepared bluntly and luxated from its compartment (Fig. 3c). Using a scalpel, the tendon is resected from the calcaneal bone at its insertion. If a bone involvement has been diagnosed, the Tuber calcanei can now be resected with a chisel, in one piece with the tendon (Fig. 3a, b, d). Cranially the tendon is resected at the point where it spreads into the aponeurosis. The resected specimen (Fig. 3e, f) is sent to histopathological examination. Microbiological probes are harvested from the wound, after which irrigation and hemostasis are performed, followed by wound closure, especially of the proximal and distal wounds (Fig. 3g). If direct wound closure is not feasible, negative pressure wound therapy (NPWT) with 125 mmHg is initiated. After infection control and wound stabilization, the wound can be closed either by split-thickness skin grafting (STSG), predicted or free flaps.

## Results

Thirty patients with an average age of 70.3 (63–87) years were included in the study. There were 11 women and 19 men in the cohort. The average body mass index (BMI) was 27.24 (standard deviation (SD) 5.97). Considering the comorbidities, most of the patients had arterial hypertension (80%), followed by diabetes mellitus (46.67%), obesity (30%) and peripheral artery disease (PAD) (10%). The American Society of Anesthesiologists physical status classification (ASA) was two in five patients, three in 23 patients and four in two patients. The right leg was involved in 43.3% (*n* = 13) patients, the left leg in 50.0% (*n* = 15) patients and 6.6% (*n* = 2) patients had a bilateral lesion. Two patients were amputated during the inpatient treatment, both having advanced peripheral arterial disease without improvement after minimally invasive intervention.

The time elapsed from the first diagnosis of Achilles tendon infection (ATI) until the admission to our clinic was 75.4 (0–1263) days. All patients finally received a complete resection of the AT, whereby in 71.88% (*n* = 23) cases the tendon was resected at the first surgery and in the remaining 28.13% (*n* = 9) during the following surgeries. The complete en bloc resection of AT (Fig. 3c–f) was performed in 62.5% (*n* = 20) of the cases, while the rest 37.5% (*n* = 12) received a gradual resection in several surgeries. When possible, partial retention of AT was intended, which was often not feasible due to the extended infection of the tendon. The patients were operated on average 4.94 times (2–13, SD 2.27).

When analyzing the etiology of the open wound which led to the ATI, we found 13 patients (43.3%) to have a chronic leg ulcer, six patients (20%) had received an open AT repair, another six patients had chronic wounds after minor trauma and the remaining five patients (16.7%) had traumatic degloving, resection of a squamous cell carcinoma (Fig. 3), pressure sore and two Pyoderma gangrenosum. Five patients had an associated osteomyelitis of the calcaneus.

**Fig. 4:**
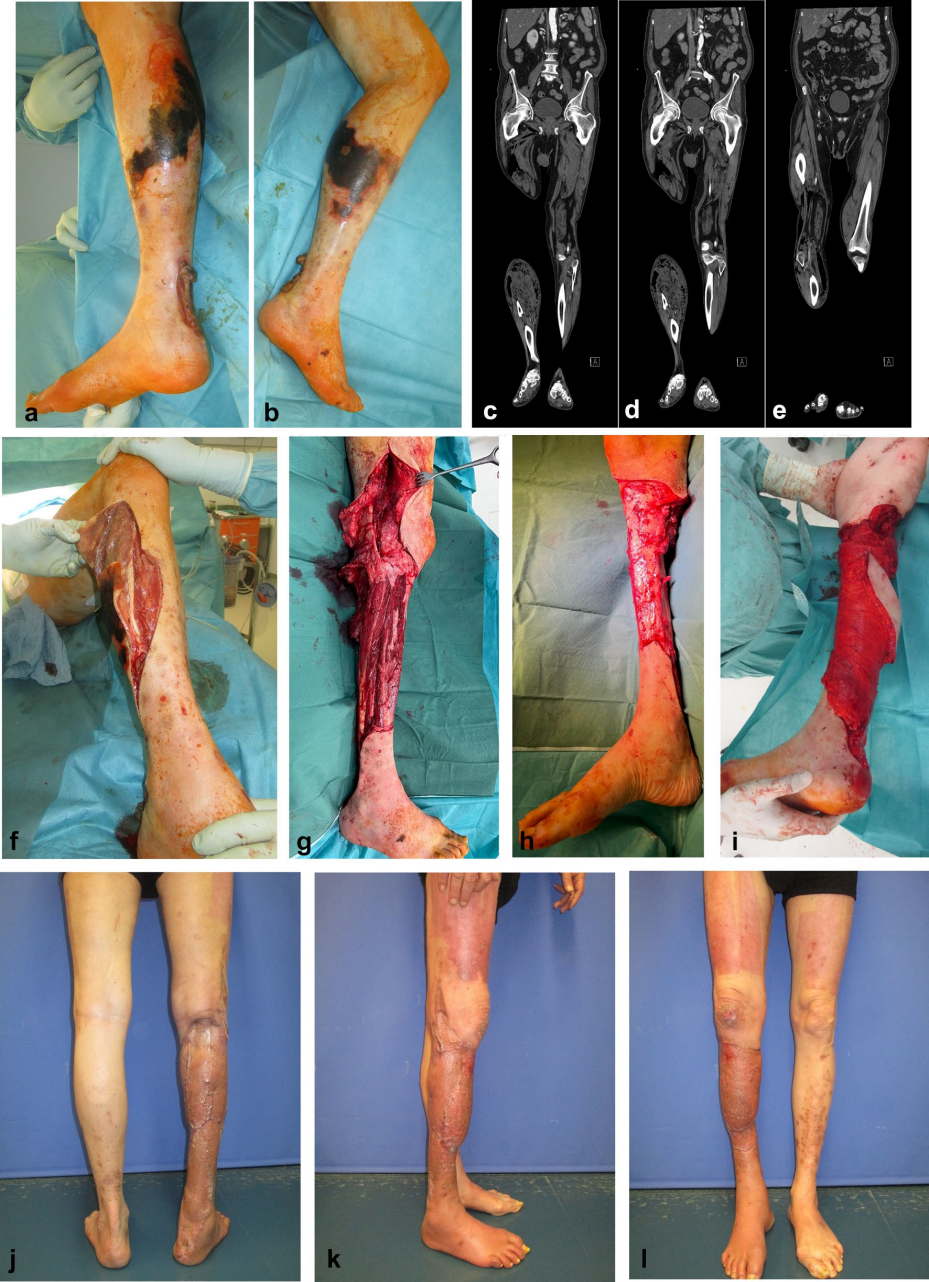
Case report 2: 63-year-old male with necrotizing fasciitis on top of a neglected AT wound after a minor injury. **a** Initial clinical finding, medial view. **b** Initial clinical finding, lateral view. **c**, **d**, **e** CT scan depicting air pockets from the leg up to the thigh and gluteal region. **f** Abscess formation around the muscle fascia of the leg. **g** Radical debridement; the necrotic muscle fascia was resected together with the complete necrotic triceps surae muscle and AT; finally, the wound was packed in antiseptic bandages; the patient recovered fast and was transferred to a normal ward; empiric antibiotic therapy was initiated and later adapted to the antibiograms. **h** Follow-up surgery with clean wounds and exposed anterolateral tibia; NPWT was initiated. **i** Free latissimus dorsi flap transferred for soft tissue coverage. **j**, **k**, **l** Final result 4 months after initial admission

**Fig. 5:**
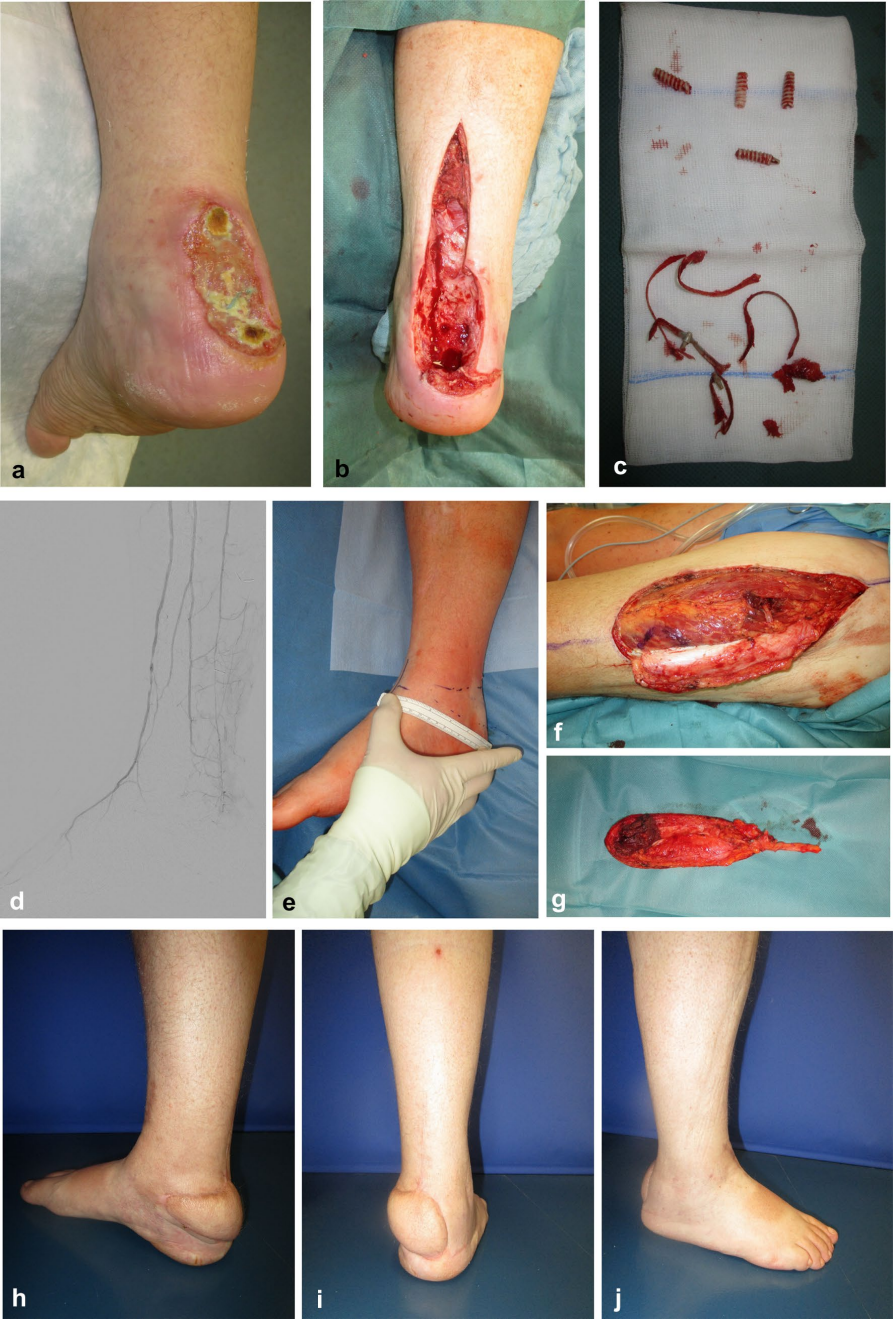
Case report 3: 59-year-old patient with peripheral arterial occlusive disease after AT repair with bone refixation. **a** Initial clinical finding. **b** Intraoperative finding after tendon resection. **c** Suture material and bone anchors retrieved from the calcaneus. **d** Digital subtraction angiography showing that the leg perfusion was ensured only by the anterior tibial artery, which was itself calcified. **e** Flap planning; an end-to-side anastomosis to the dorsalis pedis artery was foreseen. **f** Anterolateral thigh flap preparation from the contralateral side. **g** The harvested flap with a muscle cuff to fill the calcaneal bone defect and with eccentrically placed perforator vessels, to reach the recipient vessels on the back of the foot. **h**, **i**, **j** Outcome at two-year follow-up; the flap has been liposuctioned once to allow the foot to fit in normal footwear; the patient works again as a taxi driver

Considering the closure technique, four wounds could be closed primarily, 23 wounds were treated with NPWT and STSG, two patients received a free latissimus dorsi flap and STSG (Fig. 4) and one patient received a free anterolateral thigh perforator flap (ALT) (Fig. 5). No ATR was attempted. In the 28 patients where the legs could be saved, 14 wounds were completely closed at discharge and 12 patients had small residual wounds (< 1 cm), which healed ambulatory. Two patients were discharged with larger residual wounds (> 1 cm) and received further outpatient wound care. The treatment time in our clinic was on average 37.8 days (6–95, SD 21,47).

**Fig. 6:**
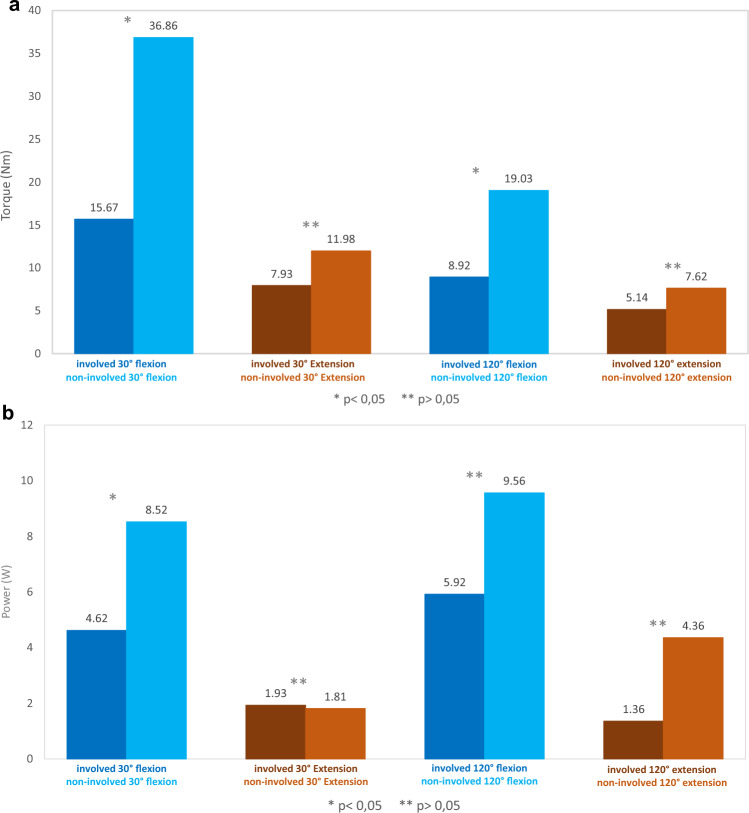
**a** Torque (Nm) of both legs for flexion and extension. **b** Power (W) of both legs for flexion and extension

The re-evaluation was performed on 14 patients, with a mean follow-up time of 19 months (4–49, SD 12.6). Using the IsoMed 2000® device, two examinations were performed, each with three repetitions. After choosing the better values from the two examinations, an average was made from the values of the three repetitions. Finally, we obtained values for the absolute maximum torque in Nm and the power in Watt, which were statistically evaluated. As expected, the absolute maximum torque was attenuated on the operated side compared to the healthy side, not only for the ankle flexion, but also for the extension. The 30°/s measurement showed the greatest difference, with an average loss in flexion torque of 57.49% on the injured side (*p* = 0.003) and an average loss in extension torque of 33.81% (*p* = 0.069) compared to the healthy side. With the 120°/s measurement the differences between the sides were less pronounced, with a loss in torque on the operated side of 53.13% (*p* = 0.05) for flexion and 32.55% (*p* = 0.075) for extension compared to the healthy side (Fig. 6a).

The loss in power on the operated side compared to the healthy side for flexion at 30°/s was 45.77% (*p* = 0.025) and at 120°/s was 68.81% (*p* = 0.423), while for an extension at 30°/s the residual power was on average 6.63% more on the injured side than on the healthy side (*p* = 0.877), while at 120°/s the power was 68.81% less on the injured side (*p* = 0.292) (Fig. 6b).

**Fig. 7:**
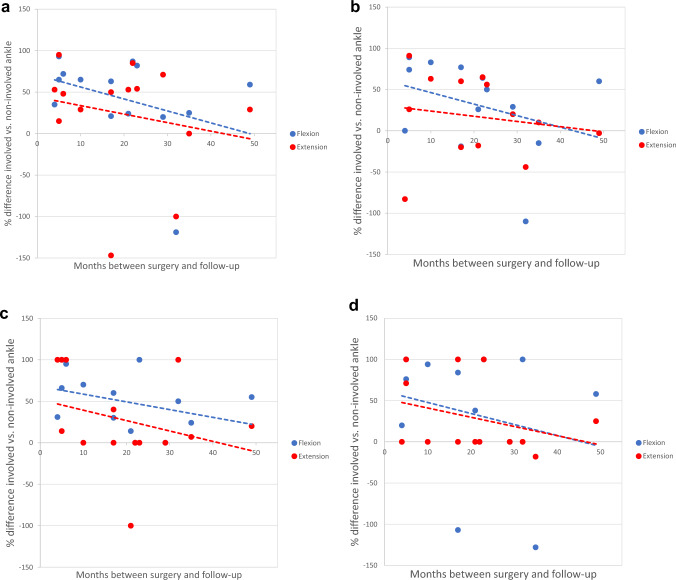
**a** Torque (Nm) at 30°/s in relation to time. **b** Torque (Nm) at 120°/s in relation to time. **c** Power (W) at 30°/s in relation to time. d Power (W) at 120°/s in relation to time

As the follow-up time ranged from 4 to 49 months, we searched for a correlation between the time elapsed from surgery and ankle joint strength. We found a tendency toward the reduction of strength difference between the operated and healthy sides, for flexion and extension, although without a statistical significance (Fig. 7a–d).

**Fig. 8:**
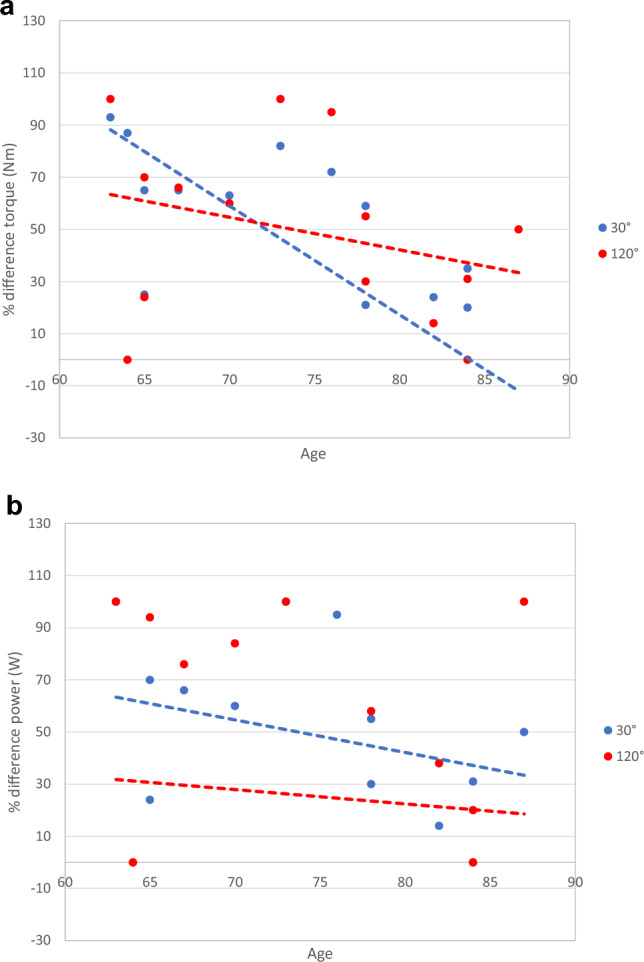
**a** Age in relation to torque. **b** Age in relation to power

The age of the re-evaluated patients varied between 63 and 87 years old. We analyzed the relation between the age of the patient and the strength of the ankle joint flexion and found that the loss in torque on the injured side increases with age at 30°/s (*p* = 0.001) and 120°/s (*p* = 0.009) (Fig. 8a). This correlation was weaker when analyzing the loss in power at 30°/s (*p* = 0.242) and 120°/s (*p* = 0.839) (Fig. 8b).

**Fig. 9:**
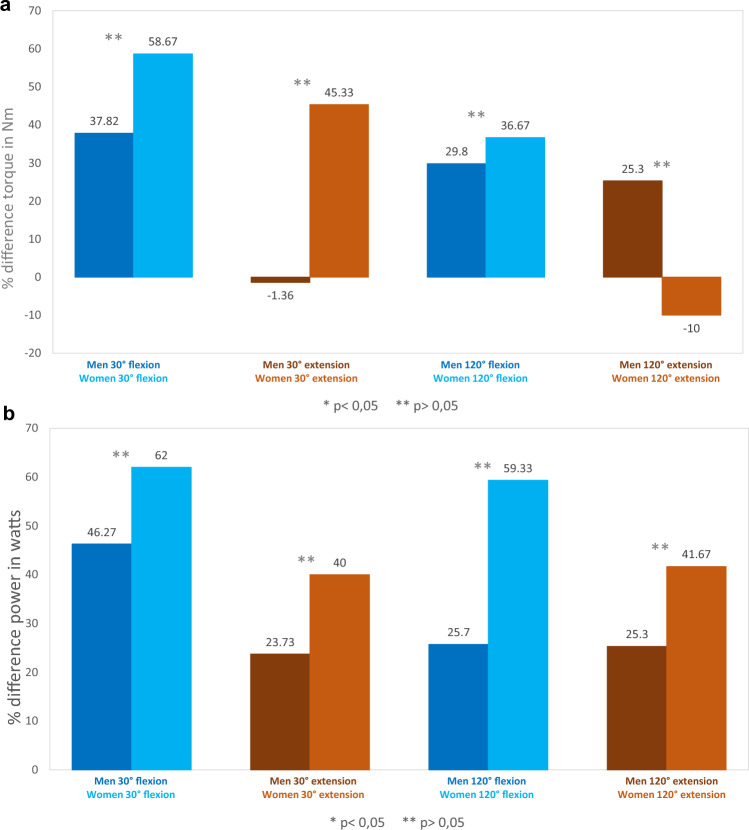
**a** Torque (Nm) in relation to gender. **b** Power (W) in relation to gender

Three women and 11 men presented for reevaluation. We compared the difference in strength between the two sexes and found that men had less torque and power loss with flexion in the injured leg and had on average more strength with extension in the injured leg than the healthy side (Fig. 9a, b). Considering the smaller female group (three patients), statistical significance could not be evaluated.

**Fig. 10:**
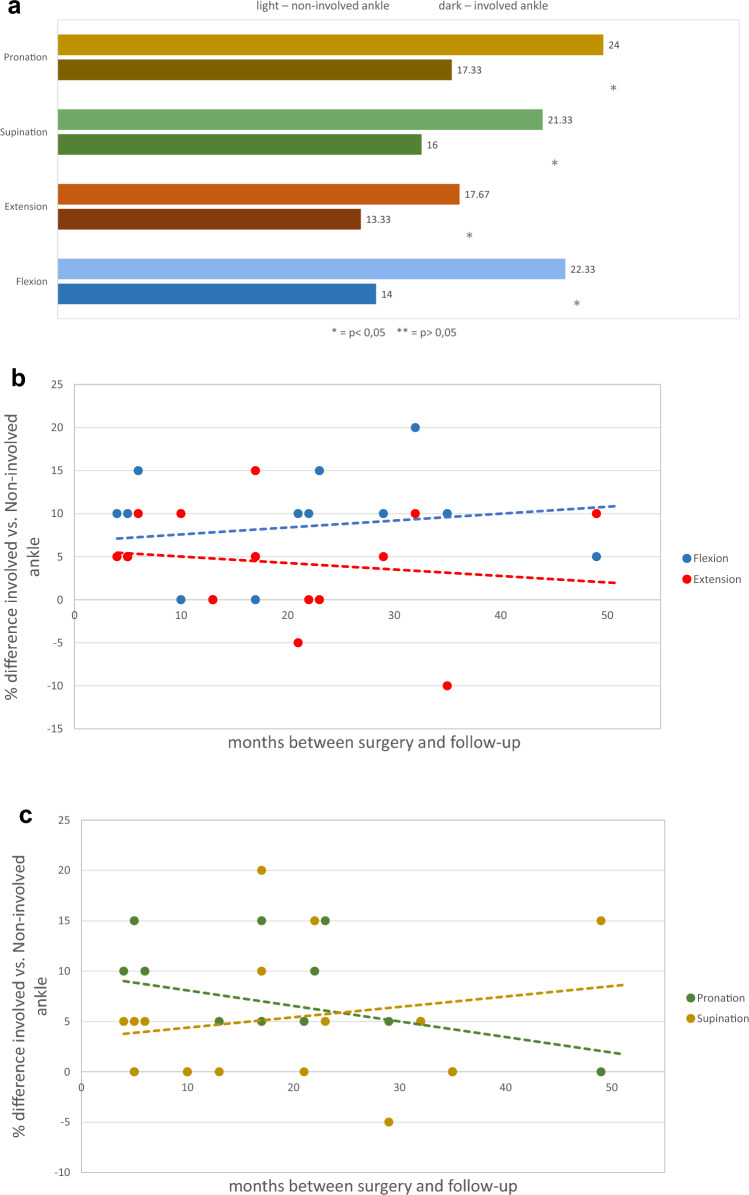
**a** Ankle Range of Motion. **b** Range of Motion in relation to time. **c** Range of Motion in relation to time

There was a statistically significant loss of range of motion on the operated side compared to the healthy side: 37.30% (22.33° vs 14°), for plantar flexion (*p* = 0.003), 24.56% (17.67° vs13.33° for dorsal extension (*p* = 0.014), 27.79% (24° vs 17.33°) for pronation (*p* = 0.002), and 24.99% (21.33 vs 16°) for supination (*p* = 0.037) (Fig. 10a). When considering the follow-up time, the flexion and supination showed an improvement with elapsed time, while the extension and pronation showed a deterioration, without statistical significance (Fig. 10b, c).

**Fig. 11:**
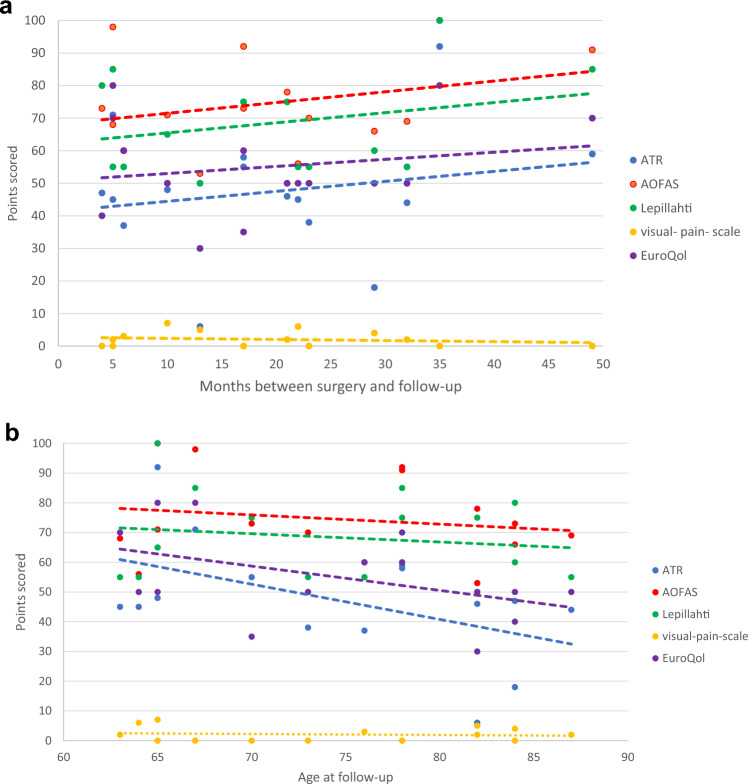
**a** Scores in relation to time. **b** Scores in relation to age

Table 1 depicts the raised functional scores. There was a trend toward better functional scores with longer follow-up times (Fig. 11a) and a trend toward worse functional scores with growing age (Fig. 11b), without statistical significance. The gait was evaluated within the AOFAS score. Five patients had a normal gait, seven patients could walk almost normally with walking support and three patients needed a wheelchair to cover longer distances. The patients with normal gait had less strength difference between the legs and were younger.

## Discussion

In this study, we could show that the radical resection of the infected AT leads to control of the infection and successful wound closure. A satisfying residual function in the ankle joint remains, which offers the patients a good quality of life.

We could show in 30 patients that ATI is a serious complication that can have dramatic consequences if not appropriately treated. Although many practitioners are reserved in sacrificing the AT, we showed that the advantages of achieving infection control and wound closure outweigh the functional impairment. Especially in older, morbid patients, prompt AT resection and direct or STSG wound closure may save the patient’s limb and even life.

The functional evaluation showed that a significant drop in torque and power for plantar flexion occurs on the injured leg compared to the healthy side. Furthermore, there was a loss in extension torque and power on the injured side. This could reflect on one hand a functional disbalance of the leg flexors and extensors but on the other side, it could also reflect the prolonged treatment in these patients, which implied reduced mobility with muscle atrophy and function loss. The evaluation according to the follow-up time showed a trend toward strength improvement, which could be explained by the muscle hypertrophy in the deep posterior muscle compartment but also by the rehabilitation and physical exercise which led to improved mobility and gait. We could also show that the patient’s age is an important factor for functional recovery. The younger the patient, the better the expected result. Considering all these data, we can conclude that the outcomes are more than satisfying in a cohort aged 70.3 years on average. The scores confirm the good functional results while showing a low residual pain level. The lower EurQol score of 55 from 100 reflects not only the leg function but also the general condition of the patient due to their age and comorbidities.

Situated in a superficial position, the AT is easily exposed when the skin and the subcutaneous tissues are breached. This may happen through sharp trauma, leg ulcers due to vascular impairment (venous, arterial, mixed), skin necrosis due to pressure, friction, infection, dermatological diseases or iatrogenic after open AT repair[3, 4, 8]. The peritendineum offers nutrition and protection to the voluminous bradytroph tendon tissue [1]. When this sheath is breached, the contamination of the tendinous tissue needs to be promptly addressed to save the tendon. Especially foreign bodies, including suture material and bony fixation devices, form a biofilm and sustain the infection and therefore need to be promptly debrided and removed [5]. The development of NPWT has dramatically improved the treatment of chronic wounds and is a valuable tool in closing leg wounds and preserving the injured AT [5]. The percutaneous AT repair offers the advantage of maintaining the synovial blood supply while avoiding the problematic wound over the tendon. Studies have shown that this technique may reduce the incidence of wound complications and AT infections [9]. Despite all these surgical advances, the exposed AT may become imbibed and diffusely contaminated, at which point a complete resection is not avoidable. In these situations, a partial AT resection would leave contaminated necrotic tissue in place and maintain the infection indefinitely. Especially in elderly, multimorbid patients, this can have dramatic consequences, including abscess formation, necrotizing fasciitis, limb loss and even death (Fig. 4a, 4b). The radical debridement must include, therefore, all the necrotic tissues, the whole contaminated tendinous tissue, all the foreign bodies, including bone anchors and possibly the necrotic bone fragments in the calcaneus (Fig. 5a, b, c). If calcaneus osteomyelitis is diagnosed in the MRI, then a six-week antibiotic cure should be initiated.

There are different ways for ATR. If there is no soft tissue defect, an FHL tendon transfer can be performed. This changes the insertion of the FHL from the base of the distal phalanx of the hallux to the distal Achilles tendon or Tuber calcanei. In a study on 11 patients with chronic AT rupture treated with FHL tendon transfer, Wegrzyn et al. found a loss of torque of 28 ± 11% at 30°/s and 36 ± 4% at 120°/s compared to the opposite side at 79 months average follow-up [10]. A similar solution is the transfer of the peroneus brevis tendon, with comparable results to the FHL tendon transfer [11, 12]. Another alternative is the FDL tendon transfer, whereas no studies are comparing it with the FHL transfer [5]. One should consider nevertheless that these techniques can be associated with wound healing complications [11], that tendon transfer with suture material and possibly bone anchors suppose a high infection risk in a septic situation and should be avoided whenever possible and that the final loss of flexion strength is not negligible. Tendon autografts, allografts, xenografts or synthetic grafts can also be used to bridge the tendon gap [11]. The prerequisite is nevertheless a closed wound without bacterial contamination. In septic cases, a vascularized tendon transfer is preferred to avoid infection relapse, loss of graft and development of osteomyelitis [4].

If there is a soft tissue defect, a plastic surgeon should be involved. While classically the so-called reconstructive ladder has been applied, where the surgeon gradually deploys the simplest available solution for wound closure [4], the modern plastic surgery flap employs the reconstructive elevator [13] or clockwork [14], where the most appropriate combination of reconstructive techniques is applied to achieve an optimal result. In AT septic surgery, closure with remote tissue containing a vascularized tendon would be the ideal solution. This brings well-vascularized soft tissue and tendon for wound healing as well as robust skin and subcutaneous tissue to resist the shear forces in this area and avoid inducing more trauma through the donor site to an already injured leg. Several factors have to be taken into consideration like comorbidities, local vessel status and patient availability to sustain prolonged surgery and rehabilitation, before taking the reconstructive elevator straight to the highest floor, free flaps.

In elderly, multimorbid patients, like in our cohort, a swift, uncomplicated wound closure might serve the best patient. Therefore, after achieving infection control and a clean wound, the direct closure over drainages or STSG with additive NPWT might best serve the goal [5]. A troublesome region is the tuber calcanei, which usually can be closed directly after tendon resection. If this is not the case and the bone is exposed, prolonged NPWT with the addition of dermal substitutes may help achieve a granulating wound for skin transfer. When the calcaneal bone has been debrided and is exposed, a free flap might nevertheless remain the only option for limb salvage (Fig. 5 a–j). The main disadvantage of skin grafting is that the scar tissue is prone to breakdown due to friction with clothing and footwear, so careful long-time scar care is necessary. Also, a secondary tendon reconstruction would be difficult after split thickness skin grafting. Fourniols et al. published a series of 15 ATI with radical AT resection and secondary wound healing. Although they report good results and the formation of a neo-tendon on MRI due to the scar tissue, the mean wound closure time was 61 days [6].

Local flaps are a straightforward solution. The distally based fasciocutaneous sural flap is a common solution for covering the AT. It offers abundant, thin tissue with effortless dissection. The donor site morbidity, lack of available vascularized tendon and the relatively high complication rate of 26.4%, especially in the elderly and in patients with venous insufficiency render it a backup solution in our practice [15–17].

Free flaps play a key role in ATR. Out of the fasciocutaneous flaps, the lateral arm flap, harvested with a strip of triceps tendon [23], the radial forearm flap with flexor carpi radialis tendon [24], the infragluteal flap[25], the tensor fascia lata flap [26] and ALT flap [27, 28], both harvested with a vascularized strip of fascia lata offer healthy well-vascularized soft tissue and tendon which can improve the healing of a chronically infected, poorly vascularized wound. In a systematic review, Iorio et al. report on 44 ATR with free composite grafts from 15 studies, out of which 30 vascularized tendon reconstructions and 7 avascular reconstructions. The average age of the patients was 33 years and the etiology of the defect was trauma, spontaneous tendon rupture and tumor resection. Only 50% of the patients had an infection. They found an average strength deficit of 32.3% at 30 to 60°/s and 31.5% at 120°/s for vascularized tendons and 21.2% and 24.8% for avascular ATR. The ROM was 80% for vascularized and 82% for avascular reconstructions from the contralateral side [29].

Free muscle flaps have also been employed in ATR. While the latissimus dorsi flap is ideal for covering extended leg wounds (Fig. 4h, i), the fibrosing of the muscle may transform it into a tendon-like structure [30].

Our cohort’s average age was 70.3 years, with a high incidence of comorbidities. Although we found no association between the patient-related and surgery-related risk factors and the functional outcome, most of these patients had a diminished general condition even before the Achilles tendon resection. As the patients were already hospitalized when they were admitted to our institution, the activities of daily living could not be evaluated before the treatment. The ASA scores, the comorbidities and the 20% mortality between discharge and the study begin reflect nevertheless the patients’ general condition. The postoperative functional values of 50–60% strength loss and 25–35% ROM loss compared to the healthy side were comparable to the values reached after tendon reconstruction, especially if we consider the patients’ age and functional demands.

Bersotti et al. showed in a study on 50 healthy elderly women (68.0 ± 4.6 years old) and 50 healthy elderly men (72.7 ± 8.5 years old) that torque for plantarflexion and dorsiflexion was higher in males than females at this age. Our results showed a similar trend in the injured and the healthy legs, whereas we only could examine three females in our group. Gender should also be considered when planning treatment strategies [31].

Boorboor et al. published in 2006 the only study so far about the remaining function after Achilles tendon resection [3]. They evaluated seven patients, two females and five males, who received a radical resection of the Achilles tendon without reconstruction. The infection complicated the open tendon suture in five cases and two cases were caused by leg ulcers. They used similar wound closure techniques as in our study, including direct suture, STSG and local and free flaps. The follow-up time was on average 11.4 months (5–25), shorter than 19 months in our study. The findings showed an acceptable to good gait function, with a loss in ROM on the operated side compared to the healthy side of 9° on average for dorsal extension and plantar flexion and of 16° on average for pronation and supination, values comparable to our study. The average AOFAS Score was 84.7, higher than in our study. This could be attributed to the smaller size of the cohort and the average age of the patients, 57.3 compared to 70.3 years in our study. The isokinetic strength measurements were performed at 60°/s, 120°/s and 180°/s. They found a loss of torque on the operated side compared to the healthy side of 42% at 120°/s compared to 53.13% at 120°/s in our study. Likewise, the loss of power at 120°/s was 37.2% compared to 38.08% at 120°/s in our study. Considering the differences in the cohorts, we could conclude from both studies that the remaining ankle flexion strength is between 40 and 50% from the healthy side, whereas age and comorbidities play an important role. The dorsal extension showed in their study an increase in torque and power, whereas our patients showed on average a decrease in these values. In addition to our work, they could perform a postoperative MRI on three patients, which confirmed the expectations that the triceps surae muscle atrophies, while the muscles from the deep posterior compartment hypertrophy.

The results of our study are limited and are relevant especially for the elderly patients with multiple comorbidities, which is also the population who usually develops an AT infection. The patients were recruited after the treatment was over, which is a limitation of the study, while the functional reexamination of the patients was performed in a prospective manner. A direct comparison between AT resection with and without tendon reconstruction could not be performed in our study and should be the goal of future studies, preferably in a prospective setting.

## Conclusion

In the case of an AT infection, the surgeon should thoroughly evaluate the risk–benefit ratio for the patient. As most of these conditions occur in multimorbid patients, the radical resection of the tendon with straightforward wound closure appears to be the optimal option. We could show in the current study that the complete ATR leaves the patient with an acceptable leg function and an almost normal gate while ensuring infection control and enabling wound healing. Considering the satisfactory functional results and quality of life, one would expect that most patients will be satisfied with the outcome and not wish for further treatment. If nevertheless, the active patient should wish a further functional improvement, the FHL transfer or an avascular or vascularized tendon graft may be performed after definitive wound healing, without the high risk of infection relapse in the acute situation. According to the current evidence, the patient needs nevertheless to be informed that the expected improvement is not substantial and that a 20–40% functional deficit will persist, compared to the healthy side. Hereof, further prospective studies should compare the ankle function and gait in patients with and without Achilles tendon reconstruction after complete resection.

## Data Availability

The datasets analyzed during the current study are available from the corresponding author upon reasonable request.
